# Relative Exchangeable and Exchangeable Copper: Emerging New Biomarkers for Diagnosis and Therapy Monitoring in Wilson's Disease

**DOI:** 10.1111/liv.70666

**Published:** 2026-05-04

**Authors:** Sebastian Köhrer, Antoan Rusev, Holger Zimmer, Silke Wolf, Jessica Langel, Andrea Langel, Thomas Longerich, Patrick Michl, Moritz Niesert, Alexander Fichtner, Isabelle Mohr

**Affiliations:** ^1^ Internal Medicine IV, Department of Gastroenterology University Hospital Heidelberg Heidelberg Germany; ^2^ Internal Medicine I, Department of Endocrinology, Diabetology, Metabolic Diseases and Clinical Chemistry University Hospital Heidelberg Heidelberg Germany; ^3^ Institute of Pathology, Heidelberg University Hospital Heidelberg Germany; ^4^ Department of Pediatrics I, Center for Pediatrics and Adolescent Medicine, Medical Faculty Heidelberg University Hospital Heidelberg, Heidelberg University Heidelberg Germany

**Keywords:** acute liver failure, exchangeable copper, relative exchangeable copper, Wilson's disease

## Abstract

**Background and Aims:**

Wilson's disease (WD) is a genetic disorder of copper metabolism in which early diagnosis remains challenging, particularly in acute liver failure (ALF). Relative exchangeable copper (REC) and exchangeable copper (CuEXC) are emerging biomarkers for diagnosis and monitoring, but data from larger cohorts are limited. This monocentric retrospective cohort study evaluated the diagnostic accuracy of REC in WD patients, including adult ALF and assessed the utility of CuEXC during monitoring.

**Methods:**

299 paediatric/adult patients with liver disease were analysed; 215 had confirmed WD (Leipzig score ≥ 4). Clinical data, parameters of copper metabolism and liver function tests were collected. REC was analysed in the full cohort. CuEXC was evaluated longitudinally at three standardized timepoints over 12 months in treated adult WD patients and compared with 24‐h urinary copper excretion (UCE) after 48‐h treatment interruption.

**Results:**

REC demonstrated excellent diagnostic accuracy with an AUC of 0.955 (sensitivity 88.4%, specificity 91.7%). In adult ALF, REC perfectly discriminated WD (*n* = 2/2, 100%) from other causes. CuEXC declined significantly in therapy‐naïve patients and differentiated these from very stable patients, but showed limited discrimination between stable versus unstable patients during median follow‐up. UCE off‐treatment showed parallel trajectories to CuEXC.

**Conclusions:**

REC is a highly accurate diagnostic biomarker for WD, including adult ALF. CuEXC is useful to characterize initial treatment response and copper control, but its role as a routine monitoring biomarker between stable and unstable patients remains uncertain and requires prospective validation, whereas UCE remains as a robust follow‐up parameter in these patients.

## Key Points

- REC shows excellent diagnostic accuracy (AUC 0.955) for Wilson's disease across paediatric and adult patients.
- REC reliably identifies WD‐related acute liver failure in adults, though larger confirmational studies are needed.
- CuEXC declines after initiation of chelation therapy and differentiates therapy‐naïve from very stable patients.
- UCE off‐treatment remains the guideline‐endorsed monitoring standard; CuEXC needs further validation for borderline stable/unstable scenarios.

## Lay Summary

Wilson's disease (WD) is a rare genetic copper disorder mainly affecting the liver. This retrospective study shows a blood test called relative exchangeable copper (REC) accurately diagnoses WD in children and adults, including emergency cases with acute liver failure. Newer blood tests (exchangeable copper) track early treatment very well and identify very stable patients, but need more validation for routine use. For copper monitoring in stable/unstable patients, 24‐h urine copper testing remains most reliable.

**Abbreviations**
24 h‐UCE: 24‐h urinary copper excretion
ALF: Acute liver failure
ALI: Acute liver injury
AUC: Area under the curve
Cp: Ceruloplasmin
Cu: Copper
CuEXC: Exchangeable copper
DPA: D‐penicillamine
EASL: European Association for the Study of the Liver
INR: International normalized ratio
IQR: Interquartile range
KFR: Kaiser‐Fleischer ring
LFT: Liver function test
MASLD/MASH: Metabolic dysfunction‐associated steatotic liver disease/steatohepatitis
MRI: Magnetic resonance imaging
NCC: Non‐ceruloplasmin copper
PFIC: Progressive familial intrahepatic cholestasis
REC: Relative exchangeable copper
ROC: Receiver operating curve
ULN: Upper limit of normal
WD: Wilson's disease

## 1 Introduction

Wilson's disease (WD) is an autosomal recessive disorder of copper (Cu) metabolism resulting from various mutations in the ATP7B gene [1, 2, 3]. Cu excess primarily harms the liver and the brain, resulting in a variety of symptoms [4]. WD is treated with chelators (D‐penicillamine (DPA), trientine) or zinc salts to achieve a negative copper balance and stabilize copper homeostasis [5, 6, 7, 8]. Current guidelines recommend a Leipzig Score ≥ 4 for the diagnosis of WD. This score combines clinical and laboratory findings: neurological symptoms and/or presence of Kayser–Fleischer rings (KFR); abnormal brain magnetic resonance imaging (MRI); elevated hepatic Cu content; laboratory abnormalities, such as hemolytic anaemia, low serum ceruloplasmin (Cp), increased urinary Cu excretion (UCE); and the identification of pathogenic ATP7B variants [9, 10]. Assessment of copper metabolism is crucial for both diagnosis and therapeutic monitoring in patients with WD. NCC (non‐ceruloplasmin‐bound copper) has long been recommended for monitoring, proposed to reflect so‐called ‘free’ copper. However, NCC is calculated via a method that is dependent on ceruloplasmin. NCC is therefore considered a less reliable parameter than 24 h‐UCE [11, 12, 13, 14]. Instead, the free copper fraction can be determined via ultrafiltration coupled with atomic absorption spectrometry. This ‘free’ or exchangeable copper (CuEXC) is a new biochemical marker of copper overload independent of ceruloplasmin [4, 15, 16]. High CuEXC values (especially > 2.08 μmol/L) are associated with extrahepatic involvement and disease severity [17]. Additionally, the ratio of CuEXC to total serum copper, termed ‘relative exchangeable copper’ (REC), has been shown to enable diagnosis of WD with a high sensitivity and specificity employing a cut‐off value > 15% [18, 19, 20], as also demonstrated by Guillaud et al. [21]. The new guideline of the European Association for the Study of the Liver (EASL) therefore acknowledged REC as an additional diagnostic test to confirm diagnosis of WD [10]. With exception of the two French WD register and a combined Danish‐Spanish cohort, REC has not been validated in larger cohorts yet [19, 20, 21]. REC has been demonstrated helping to identify patients with acute liver failure due to WD in a paediatric cohort [22, 23], but this has not been proven in adults yet. Moreover, CuEXC might be a future biomarker for frequent monitoring of copper metabolism, which has been demonstrated in a paediatric cohort, revealing a significant decrease during the first year of treatment, similar to UCE [24]. In contrast, a Spanish adult cohort study raised doubts on the utility of CuEXC, as it performed as a suboptimal tool for monitoring Cu metabolism during short and median follow‐up [25]. Further data in adults and long‐term data are still lacking. This study aims to re‐evaluate the diagnostic potential of REC and other markers of copper metabolism and to assess their usefulness in diagnosing WD in both paediatric and adult patient cohorts in Germany with a special consideration of adult patients with acute liver failure. In addition, CuEXC was analysed in comparison with UCE and NCC for monitoring WD in adult patients, with a focus on its suitability for short‐, median‐ and long‐term follow‐up.

## 2 Materials and Methods

### 2.1 Study Population

All patients presenting to the outpatient clinic and/or the intensive care unit of the Department of Gastroenterology or Department of Paediatrics at Heidelberg University Hospital from January 2024 to August 2025 for whom REC data were available were considered for this study. For all patients, clinical parameters, laboratory results including copper parameters (REC, CuEXC, total copper, NCC, Cp, 24 h‐UCE) and their individual medical history including WD treatment and treatment duration were assessed. Of note, UCE was determined after 48‐h treatment cessation in our adult WD cohort. The included patients comprised patients with confirmed WD under chelation treatment, therapy‐naïve confirmed WD patients and patients with initially unknown liver disease (*n* = 84) for whom exclusion of WD as the underlying condition had to be performed. To distinguish WD from non‐Wilsonian patients, the Leipzig score was used, and a Leipzig score ≥ 4 led to the definitive diagnosis of WD according to recent guidelines [10, 26].

Further, diagnostic performance of REC in adult patients presenting with acute liver failure (ALF) was analysed. For that, all adult patients (age > 18 years) with available REC values presenting with ALF to the intensive care unit at the Department of Gastroenterology at Heidelberg University clinic between January 2024 and August 2025 were included. ALF was defined according to recent EASL guidelines [27] as new‐onset jaundice and coagulopathy (INR > 1.5) with no history of liver disease and subsequent grade ≥ II encephalopathy developing up to 28 weeks after the onset of jaundice. Patients presenting with acute‐on‐chronic liver failure (ACLF) with known preexisting liver disease were hence excluded from this study. To evaluate the performance of CuEXC as a monitoring parameter, all adult patients with confirmed WD were considered. Three different timepoints were defined for the assessment of CuEXC values over the course of the disease. The first available timepoint at which CuEXC was available was termed t0, t1 was 6 ± 1 months after t0, and t2 was 12 ± 1 months after t0. Patient stability was determined using a composite definition incorporating clinical status, liver function tests (LFTs) and UCE measured off‐treatment after 48 h therapy cessation, per guidelines [10] and prior literature [13, 14]. UCE target ranges for treated WD adults were: optimal control < 1.5 μmol/d, acceptable 1.5–2.5 μmol/d, poor control > 2.5 μmol/d. Patients were stratified as: therapy‐naïve (no prior anti‐copper therapy, *n* = 6); very stable (*n* = 28), stable (*n* = 73) or unstable (*n* = 108). Additional conditions and definitions for grouping among these four groups are described in detail in Appendix S1. Additionally, we categorized our adult WD cohort into four subcohorts according to time of treatment: ‘therapy‐naïve’ (≤ 1 year of treatment), ‘short‐term’ (> 1–5 years of treatment), ‘median‐term’ (> 5–10 years), and ‘long‐term’ (> 10 years). CuEXC was measured using ultrafiltration‐determination, and REC was calculated according to the formula: REC (%) = CuEXC [μmol/l]/total serum copper [μmol/l] × 100 (see also Appendix S1).

### 2.2 Statistical Analysis

Data were collected in a pseudonymized database and analysed using IBM SPSS Statistics (version 31.0, IBM, Chicago, IL, USA). Descriptive statistics included frequencies and measures of central tendency and dispersion. Results are reported as median (IQR) unless stated otherwise. Group comparisons were performed using the Mann–Whitney *U* test (two independent groups), Kruskal–Wallis test (> 2 independent groups) and Wilcoxon test (paired data). Statistical significance was set at *p* < 0.05. Receiver operating characteristic (ROC) analysis was used to determine optimal diagnostic thresholds for WD for REC, Cp and total serum copper using the Youden method. Figures were generated using SPSS and Matplotlib (Python).

## 3 Results

### 3.1 Patient Characteristics

A total of 299 individuals (50.5% female) were included in the analysis, of whom 215 were diagnosed with WD (Leipzig score ≥ 4) and 84 had other hepatic disorders. 41 of the overall cohort were younger than 18 years, including 13 with confirmed WD. WD patients differed significantly from non‐Wilsonian patients across all copper metabolism parameters (Table 1): REC (*p* = 0.001), UCE (*p* = 0.001), ceruloplasmin (*p* = 0.001), total copper (*p* = 0.001) and NCC (p = 0.001). Table 2 outlines demographics and medical history of WD patients (*n* = 215). The remaining 57 non‐Wilson cases (excluding 27 patients with acute liver failure) were predominantly characterized by autoimmune hepatitis (*n* = 22), followed by drug‐induced liver injury (*n* = 10), metabolic dysfunction‐associated steatotic liver disease/steatohepatitis (MASLD/MASH) (*n* = 7), alcohol‐related liver disease (*n* = 8) and viral etiologies (*n* = 4), primary sclerosing cholangitis (*n* = 5) and progressive familial intrahepatic cholestasis (PFIC) type 2 (*n* = 1). For WD patients, the median treatment duration was 19.37 years. D‐penicillamine was used in 78 patients (36.28%), trientine in 105 (48.84%; thereof *n* = 99 received trientine dihydrochloride and *n* = 6 trientine tetrahydrochloride) and zinc salts in 26 (12.09%). Six patients (2.79%) were treatment‐naïve at inclusion and all were subsequently started on D‐penicillamine. Median daily doses of the total cohort were: for DPA, 1200 mg (IQR 900–1500 mg), for trientine dihydrochloride 1200 mg (IQR 800–1400 mg) versus for trientine tetrahydrochloride 750 mg (IQR 450–900 mg) and for zinc acetate, 150 mg (IQR 135–150 mg). 76.3% patients initially presented with hepatic symptoms, whereas 23.7% showed neurological or mixed phenotypes. In our cohort, all patients identified through family screening (*n* = 3) had at least mild biochemical or clinical abnormalities at diagnosis. Table S1 summarizes clinical and biochemical characteristics by WD phenotype. All paediatric cases presented with a hepatic phenotype. The median Leipzig score at diagnosis was 7.7 (IQR 6.50–9.40). Biallelic pathogenic ATP7B variants were identified in 169/215 patients (78.60%) and monoallelic variants in 36 (16.64%); in 10 patients, genetic data were unavailable, but WD was confirmed by histology and UCE. Liver cirrhosis was present in 26 adult patients, all classified as Child–Pugh A.

**TABLE 1:** Baseline characteristics of Wilson's disease versus non‐Wilsonian patients.

Parameter	Total cohort *n* = 299	Paediatric sub‐cohort *n* = 41	Wilsonian sub‐cohort *n* = 215	Non‐Wilsonian sub‐cohort *n* = 84	*p*
Age [years]	37.9 (26.2–50.9)	13.4 (9.2–16.1)	40.3 (29.5–53.6)	29.3 (16.3–46.9)	**< 0.001**
Sex (female in %)	151 (50.5)	21 (48.8)	107 (49.8)	41 (48.8)	0.882
Relative Exchangeable Copper [%]	16.6 (8.2–30.5)	7.3 (5.3–18.4)	20.6 (14.3–38.1)	6.2 (5.12–8.2)	**< 0.001**
Exchangeable Copper [μmol/L]	0.92 (0.67–1.22)	0.94 (0.81–1.20)	0.95 (0.68–1.27)	0.86 (0.67–1.09)	0.053
24 h Urinary copper excretion [μmol/d]	1.44 (0.66–2.22)	0.51 (0.25–1.04)	1.65 (1.02–2.37)	0.35 (0.22–1.04)	**< 0.001**
Non‐ceruloplasmin bound copper (NCC) [g/L] (calculated)	0.76 (0.19–1.89)	2.62 (0.76–4.32)	0.6 (0.16–1.49)	1.9 (0.63–3.45)	**< 0.001**
Ceruloplasmin [g/L]	0.12 (0.06–0.19)	0.25 (0.13–0.31)	0.10 (0.04–0.14)	0.24 (0.18–0.31)	**< 0.001**
Total serum copper [μmol/L]	6.7 (2.9–11.2)	13.7 (5.9–17.1)	4.9 (2.1–8.1)	12.7 (9.1–17.5)	**< 0.001**
Aspartate aminotransferase [U/L]	30.0 (21.5–52.0)	50.0 (30.5–97.0)	28.0 (20.0–38.0)	62.0 (28.0–179.0)	**< 0.001**
Alanine aminotransferase [U/L]	46.5 (29.0–82.0)	88.0 (42.0–167.5)	45.0 (29.0–63.0)	63.0 (31.5–155.5)	**< 0.001**
Gamma‐glutamyl transferase [U/L]	36.0 (23.0–64.0)	31.0 (15.5–53.0)	34.0 (23.0–54.0)	42.5 (19.75–124.75)	**0.025**
Alkaline phosphatase [U/L]	90.5 (71.0–125.0)	247 (194.0–320.0)	85.0 (69.25–110.0)	119.5 (73.75–221.50)	**< 0.001**
Serum bilirubin [mg/dl]	0.8 (0.5–1.3)	0.5 (0.4–0.8)	0.8 (0.5–1.1)	0.9 (0.5–9.2)	0.222
International Normalized Ratio	1.04 (0.9–1.1)	1.02 (0.98–1.07)	1.03 (0.99–1.08)	1.08 (1.0–1.55)	0.145
White blood count [/nL]	5.7 (4.5–7.2)	6.28 (5.02–7.90)	5.6 (4.53–6.77)	6.49 (4.45–8.98)	0.135
Platelet count [/nL]	221 (168–268)	283 (241–361)	224 (182–266)	206 (112–280)	0.141

*Note:* Baseline characteristics of the total cohort, WD versus non‐Wilsonian patients and paediatric patients are presented. Data are shown in Median (IQR) if not stated otherwise.

* *p*‐value for Wilsonian sub‐cohort versus Non‐Wilsonian sub‐cohort.

**TABLE 2:** Clinical characteristics of Wilson's disease patients.

Parameter	Numbers, medians and IQR
Number of patients	*n* = 215 (50.2% males. 49.8% females); thereof *n* = 13 paediatric cohort
Median age at diagnosis	17.41 years (IQR_25‐75_ 10.79–26.50)
Duration of therapy	19.37 years (IQR_25‐75_ 8.60–30.43)
Recent WD‐specific therapy	D‐Penicillamine *n* = 78 (36.28%)
	Trientine *n* = 105 (48.84%); thereof TETA 2 HCl *n* = 99 (46.0%) und TETA 4HCl *n* = 6 (2.84%)
	Zinc *n* = 26 (12.09%)
	Treatment‐naïve *n* = 6 (2.79%; thereof *n* = 2 with neurological manifestation)
Clinical presentation of WD	Hepatic *n* = 164 (76.3%; thereof *n* = 13 paediatric)
	Neurological/Mixed *n* = 51 (23.7%)
Leipzig Score (total) at Diagnosis	7.70 (IQR_25‐75_ 6.50–9.40)
Pathogenic mutation of ATP7B	2 pathogenic mutations (compound heterozygous or homozygous) *n* = 169 (78.60%)
	1 pathogenic mutation *n* = 36 (16.64%)
Kayser‐Fleischer‐Ring	*n* = 64 (29.77%)
Median liver copper [μg/g]; available for *n* = 87 patients	798 (IQR_25‐75_ 410.0–1179.0)
Liver cirrhosis	*n* = 26 (12.1%; all Child‐Pugh A stage)
Acute Liver Failure	*n* = 2 (0.9%; both > 18 years)

*Note:* Clinical characteristics of WD patients are presented. Data in brackets refer to the respective proportion of patients if not stated otherwise.

### 3.2 Diagnostic Performance of REC, Cp and Total Copper

**FIGURE 1:**
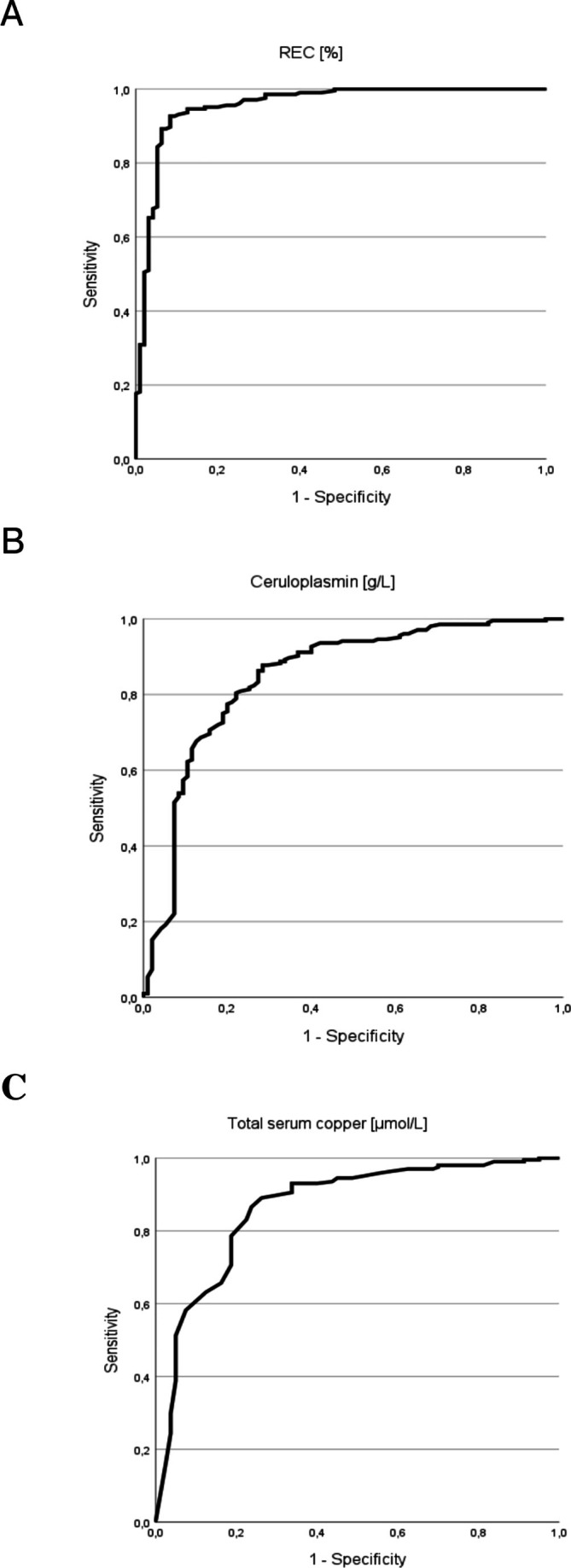
Diagnostic performance of copper metabolism parameters by ROC curve analysis. ROC curve analysis of the total study population (*n* = 299) to assess diagnostic performance of relative exchangeable copper (A), ceruloplasmin (B) and total serum copper (C). (A) AUC = 0.955, sensitivity 88.4%, specificity 91.7%, Youden index 0.800 for an optimal cut‐off of 10.6%. (B) AUC = 0.866, sensitivity 84.8%, specificity 80.0%, Youden index 0.648 for an optimal cut‐off of ≤ 0.165 g/L. (C) AUC = 0.848, sensitivity 85.6%, specificity 73.8%, Youden index 0.594 for an optimal cut‐off of ≤ 9.95 μmol/L. ROC, Receiver operating characteristic; AUC, area under the curve.

We evaluated whether REC discriminates between WD and other hepatic diseases. Given limited REC availability and the widespread use of ceruloplasmin, total copper and 24‐h UCE, these markers were also assessed as screening tests for WD by ROC analysis in the full cohort (*n* = 299; Figure 1). REC showed excellent diagnostic accuracy (sensitivity 88.4%, specificity 91.7%, AUC 0.955; Figure 1A) with an optimal cut‐off ≥ 10.6% (Youden index 0.800). All WD patients with REC values below this cut‐off were adults with biallelic pathogenic ATP7B variants diagnosed in childhood and treated with chelation therapy for > 10 years.

Ceruloplasmin showed lower sensitivity (84.8%) and specificity (80.0%) with an AUC of 0.862; its optimal cut‐off was ≤ 0.165 g/L (Youden index 0.648; Figure 1B). Total serum copper displayed slightly higher sensitivity (85.6%) than ceruloplasmin but the lowest specificity (73.8%) with an AUC of 0.842; the optimal cut‐off was ≤ 9.95 μmol/L (Youden index 0.594; Figure 1C).

### 3.3 Diagnostic Performance of REC in Patients With Acute Liver Failure

**FIGURE 2:**
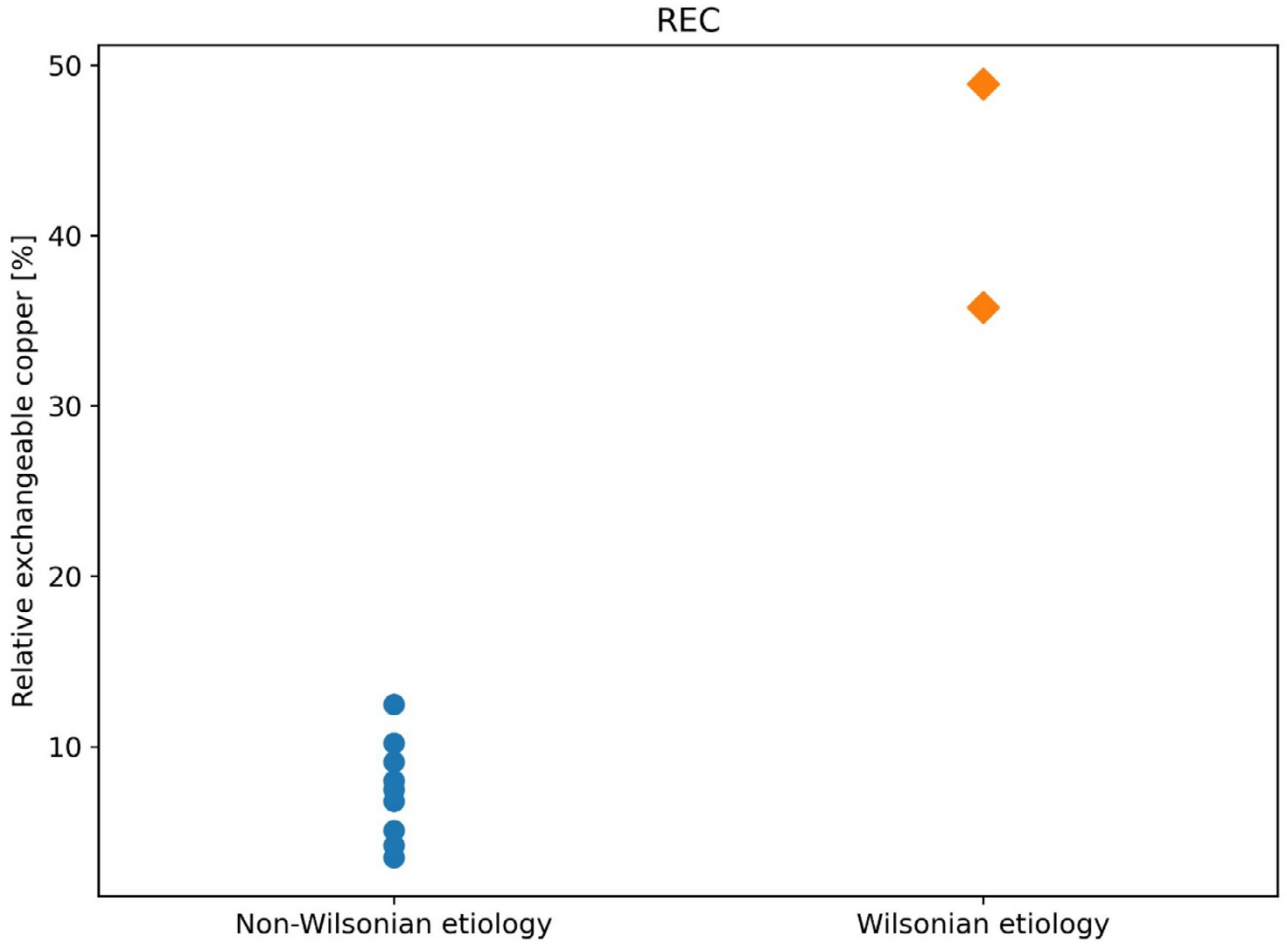
Comparison of relative exchangeable copper in adult patients with Wilson's disease‐associated versus non‐Wilsonian acute liver failure (ALF). The figure shows REC in patients with ALF due to Wilson's disease and in those with ALF due to non‐Wilsonian causes.

Diagnosing WD in patients with acute liver failure (ALF) is challenging because several parameters included in the Leipzig score are unavailable or unreliable in this setting [22]. We therefore evaluated the diagnostic potential of REC in 29 adults with ALF of unknown origin (Table 3). Among these patients, two were eventually diagnosed with WD, confirmed by strong metallothionein staining and markedly elevated hepatic copper (> 250 μg/g dry weight), and eventually genetic testing. Other ALF etiologies are stated in Table 3. Six patients (20.69%) underwent liver transplantation (including one WD case), and five (17.24%) died. Both WD patients underwent large‐volume plasma exchange after diagnosis. Detailed laboratory results are summarized in Table S2. Median REC was significantly higher in WD patients (42.2%, range 25.7%–42.2%) compared with non‐WD ALF cases (6.1%, IQR 4.4%–7.6%; *p* = 0.005; Figure 2). Median CuEXC was also significantly elevated in WD compared with other ALF etiologies (5.5 μmol/L vs. 0.76 μmol/L; *p* = 0.020).

**TABLE 3:** Clinical characteristics of patients with acute liver failure (ALF).

Parameter	Numbers, medians and percentages (in %)
Number of patients	*n* = 29 (55.18% males, 44.82% females)
Median age at diagnosis	41.50 (IQR_25‐75_ 35.62–53.58)
Aetiology of ALF	Drug‐Induced Liver Injury (DILI) *n* = 14
	Viral induced Liver Injury *n* = 3
	Cryptogenic *n* = 6
	Acute Wilson's Disease *n* = 2
	Autoimmune Hepatitis *n* = 2
	Pregnancy‐Related Liver Failure *n* = 1
	HLH Syndrome (Hemophagocytic Lymphohistiocytosis) *n* = 1
Liver transplantation	*n* = 6 (20.69%; thereof *n* = 1 with Wilson's disease)
Mortality rate	*n* = 5 (17.24%)
MELD‐Score at ALF diagnosis	29 (IQR_25‐75_ 24–36)
MELD‐Na‐Score at ALF diagnosis	27 (IQR_25‐75_ 24–30)

*Note:* Clinical characteristics of patients with ALF including clinical scores are shown. Data in brackets refer to the respective proportion of patients if not stated otherwise.

### 3.4 CuEXC as Monitoring Parameter in WD Patients Under Therapy

We next assessed whether CuEXC can serve as a monitoring marker in adult WD patients and whether dynamic changes occur during long‐term chelation therapy.

**FIGURE 3:**
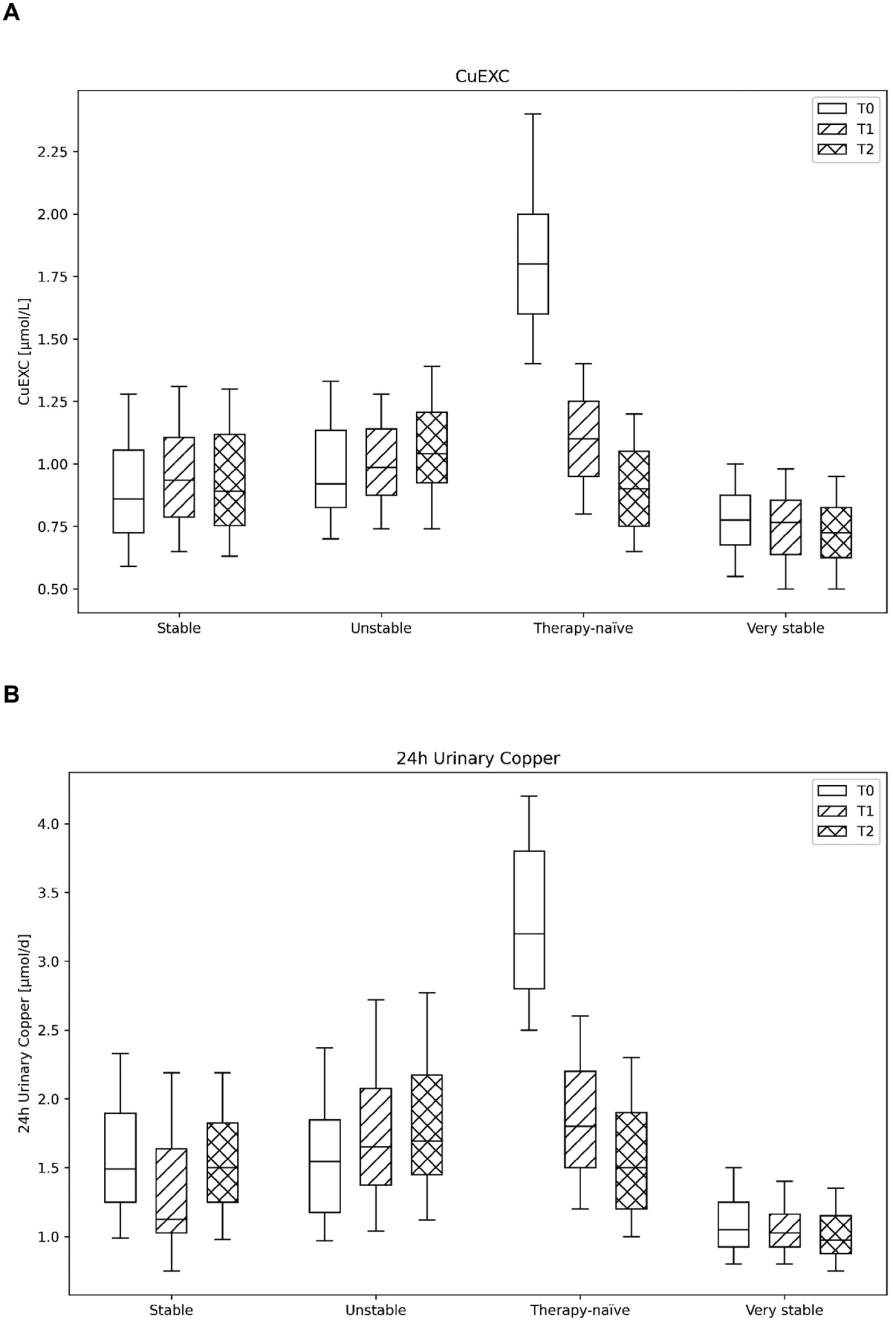
Longitudinal course of copper metabolism parameters in Wilson's disease by disease stability subcohorts. (A) Exchangeable copper, (B) urinary copper. Data presented as box plots at T0 (white fill, no hatching), T1 (white fill, diagonal hatching) and T2 (white fill, cross‐hatching) stratified by patient stability across four groups: Therapy‐naïve (no prior anti‐copper therapy, *n* = 6); very stable (*n* = 28), stable (*n* = 72) or unstable (*n* = 108) over a 12‐month observational period. Levels of significance: *p < 0.05; ns, not significant (Mann–Whitney *U* test).

Patients were categorized as ‘stable’, ‘unstable’, ‘very stable’ or ‘therapy‐naïve’ following the definitions outlined in Appendix S1. Table S3 presents median CuEXC values and corresponding daily doses according to treatment modality, clinical stability and treatment duration. CuEXC levels were generally higher in unstable than in stable patients, despite comparable or higher chelator doses in the unstable group. Furthermore, CuEXC declined progressively with longer treatment duration, with the lowest values observed in stable patients on maintenance zinc therapy. Furthermore, CuEXC declines progressively with longer treatment duration, with the lowest values observed in stable patients on maintenance zinc therapy. Among stable patients (*n* = 72), two cases showed upper‐range UCE values (Patient A: 2.33 μmol/d; Patient B: 2.39 μmol/d) but were conservatively classified as stable per composite criteria: Patient A had normal LFTs (ALT 32 U/L), no disease progression, stable therapy dose and transiently elevated UCE with lower prior/follow‐up values; Patient B exhibited mild ALT elevation (48 U/L) attributed to MASLD (biopsy‐confirmed), long‐term stability over 15 years and physician‐elected observation. These outliers highlight UCE's sensitivity within the acceptable range (1.5–2.5 μmol/d) when contextualized with clinical features. Table 4 presents laboratory parameters at t0, t1 and t2 over all groups. These targeted subgroup analyses confirm that CuEXC declines substantially during initial treatment (therapy‐naïve: 1.80 → 0.90 μmol/L, *p* = 0.005), mirroring UCE dynamics (Figure 3). However, CuEXC showed limited discriminatory capacity between stable (0.92 μmol/L) and unstable patients (0.94 μmol/L; *p* = 0.122; Table 4 and Figure 3). Additionally, we also categorized the cohort by treatment duration (Table 5 and Tables S4–S7). In the therapy‐naïve subcohort (Table S4), initiation of chelation therapy led to a statistically significant decrease in REC, CuEXC, UCE, and NCC. In parallel, liver enzymes and coagulation parameters improved. In the short‐term and median‐term subcohorts (Tables S5 and S6, respectively), overall control of copper metabolism remained stable, with no significant changes in REC and CuEXC, while UCE significantly decreased from T0 to T2. All other parameters remained largely unchanged over time and were mostly within the normal range. In the long‐term subcohort (Table S7), REC and CuEXC remained stable over time, while UCE showed a small but significant increase at generally low to moderate levels.

**TABLE 4:** Longitudinal course of copper metabolism parameters in Wilson's disease adult cohort in stable, unstable, very stable and therapy‐naïve patients.

Laboratory parameter	T0 (*n* = 180)	T1 (*n* = 180)	T2 (*n* = 180)	*p* (T0 vs. T2)
**Relative Exchangeable Copper [%]**				
Adult WD sub‐cohort	20.31 (14.22–41.72)	21.95 (14.72–36.37)	20.95 (14.25–39.25)	0.555
Stable cohort (*n* = 49/49/30)	18.0 (13.0–26.0)	18.5 (13.0–25.0)	18.0 (13.0–24.0)	0.420
Unstable cohort (*n* = 52/52/32)	22.0 (15.0–45.0)	23.0 (16.0–40.0)	22.5 (16.0–42.0)	0.380
Therapy‐naïve (*n* = 6/6/5)	45.0 (35.0–55.0)	28.0 (20.0–35.0)	22.0 (16.0–30.0)	**0.010**
Very stable (*n* = 73/73/55)	15.0 (12.0–20.0)	14.0 (11.0–18.0)	13.0 (10.0–17.0)	0.300
**Exchangeable Copper [μmol/L]**				
Adult WD sub‐cohort	0.94 (0.66–1.29)	0.99 (0.68–1.28)	0.99 (0.67–1.35)	0.122
Stable cohort (*n* = 49/49/30)	0.92 (0.59–1.28)	0.97 (0.65–1.31)	0.93 (0.63–1.30)	0.405
Unstable cohort (*n* = 52/52/32)	0.94 (0.70–1.33)	1.02 (0.74–1.28)	1.08 (0.74–1.39)	0.122
Therapy‐naïve (*n* = 6/6/5)	1.80 (1.40–2.40)	1.10 (0.80–1.40)	0.90 (0.65–1.20)	**0.005**
Very stable (*n* = 73/73/55)	0.80 (0.55–1.00)	0.78 (0.50–0.98)	0.75 (0.50–0.95)	0.400
**24 h Urinary Copper Excretion [μmol/d]**				
Adult WD sub‐cohort	1.67 (0.98–2.38)	1.56 (0.90–1.33)	1.70 (1.00–2.40)	**< 0.001**
Stable cohort (*n* = 49/49/30)	1.58 (0.99–2.33)	1.15 (0.75–2.19)	1.60 (0.98–2.19)	**< 0.001**
Unstable cohort (*n* = 52/52/32)	1.69 (0.97–1.37)	1.70 (1.04–2.72)	1.79 (1.12–2.77)	**0.012**
Therapy‐naïve (*n* = 6/6/5)	3.20 (2.50–4.20)	1.80 (1.20–2.60)	1.50 (1.00–2.30)	**0.003**
Very stable (*n* = 73/73/55)	1.10 (0.80–1.50)	1.05 (0.80–1.40)	1.00 (0.75–1.35)	0.350
**Non‐ceruloplasmin Copper [g/L] (calculated)**				
Adult WD sub‐cohort	0.66 (0.16–1.40)	0.69 (0.14–1.51)	0.41 (0.10–1.11)	**0.027**
Stable cohort (*n* = 49/49/30)	0.52 (0.01–1.40)	0.57 (0.25–1.67)	0.33 (0.01–1.10)	0.255
Unstable cohort (*n* = 52/52/32)	0.71 (0.26–1.51)	0.74 (0.03–1.40)	0.49 (0.01–1.20)	**0.041**
Therapy‐naïve (*n* = 6/6/5)	1.20 (0.80–1.80)	0.80 (0.40–1.40)	0.60 (0.20–1.10)	**0.020**
Very stable (*n* = 73/73/55)	0.40 (0.05–0.90)	0.38 (0.05–0.85)	0.35 (0.05–0.80)	0.300

*Note:* Longitudinal course of copper metabolism parameters is depicted according to disease stability. Data are shown as median (IQR) if not stated otherwise.

**TABLE 5:** Longitudinal course of copper metabolism parameters in adult Wilson's disease patients (therapy‐naïve and during short‐, median‐ and long‐term follow‐up).

Laboratory parameter	WD sub‐cohort at T0; *n* = 180	WD sub‐cohort at T1; *n* = 180	WD sub‐cohort at T2; *n* = 120	*p* (T0 vs. T2)
**Relative Exchangeable Copper [%]**	20.3 (14.2–41.7)	21.9 (14.7–36.3)	20.9 (14.2–39.2)	0.555
**Exchangeable Copper [μmol/L]**	0.94 (0.66–1.29)	0.99 (0.68–1.28)	0.99 (0.67–1.35)	0.122
Therapy‐naïve subcohort (*n* = 6/6/5)	45.0 (35.0–55.0)	28.0 (20.0–35.0)	22.0 (16.0–30.0)	**0.010**
Short‐term sub‐cohort (*n* = 10/10/9)	0.77 (0.51–1.05)	0.52 (0.39–1.05)	0.56 (0.49–1.19)	0.790
Median‐term sub‐cohort (*n* = 32/32/23)	0.84 (0.55–1.21)	0.94 (0.64–1.14)	0.73 (0.60–1.05)	0.330
Long‐term sub‐cohort (*n* = 132/132/83)	0.98 (0.73–1.32)	1.06 (0.77–1.31)	1.08 (0.80–1.37)	0.155
**24 h Urinary copper excretion [μmol/d]**	1.67 (0.98–2.38)	1.56 (0.90–1.33)	1.7 (1.0–2.4)	**< 0.001**
Therapy‐naïve subcohort (*n* = 6/6/5)	3.20 (2.50–4.20)	1.80 (1.20–2.60)	1.50 (1.00–2.30)	**0.003**
Short‐term sub‐cohort (*n* = 10/10/9)	2.09 (1.67–4.51)	0.95 (0.88–1.48)	1.04 (0.53–2.26)	**0.043**
Median‐term sub‐cohort (*n* = 32/32/23)	1.06 (0.51–2.10)	1.14 (0.75–1.89)	1.54 (0.65–2.32)	**0.002**
Long‐term sub‐cohort (*n* = 132/132/83)	1.76 (1.16–2.50)	1.69 (1.04–2.75)	1.72 (1.17–2.52)	**0.002**
**Non‐ceruloplasmin bound copper (NCC) [g/L] (calculated)**	0.66 (0.16–1.40)	0.69 (0.14–1.51)	0.41 (0.1–1.11)	**0.027**
Therapy‐naïve subcohort (*n* = 6/6/5)	1.20 (0.80–1.80)	0.80 (0.40–1.40)	0.60 (0.20–1.10)	**0.020**
Short‐term sub‐cohort (*n* = 10/10/9)	0.21 (0.01–0.74)	0.20 (0.01–1.51)	0.06 (0.01–0.38)	0.260
Median‐term sub‐cohort (*n* = 32/32/23)	0.71 (0.01–1.63)	0.66 (0.14–1.65)	0.67 (0.06–2.09)	0.609
Long‐term sub‐cohort (*n* = 132/132/83)	0.66 (0.16–1.14)	0.74 (0.14–1.48)	0.42 (0.01–1.11)	**0.016**
**Ceruloplasmin [g/L]**	0.09 (0.03–0.14)	0.1 (0.03–0.14)	0.1 (0.04–0.15)	0.400
**Total serum copper [μmol/L]**	4.9 (2.0–7.5)	4.9 (2.0–7.8)	5.1 (1.9–7.3)	0.518
**Aspartate aminotransferase [U/L]**	28.0 (21.0–37.0)	31.0 (22.0–40.0)	34.0 (24.8–46.3)	0.435
**Alanine aminotransferase [U/L]**	45.0 (30.0–64.0)	43.0 (29.0–69.0)	45.5 (33.8–79.2)	0.556
**Gamma‐glutamyl transferase [U/L]**	36.0 (25.0–55.2)	36.0 (24.0–55.0)	38.0 (24.0–61.0)	0.456
**Alkaline Phosphatase [U/L]**	83.0 (68.0–106.0)	86.0 (71.0–114.0)	92.0 (76.5–110.3)	0.678
**Serum bilirubin [mg/dL]**	0.76 (0.57–1.06)	0.80 (0.56–1.0)	0.82 (0.56–1.13)	0.345
**International normalized ratio**	1.03 (0.99–1.08)	1.04 (1.0–1.09)	1.05 (1.0–1.1)	0.456
**White blood count [/nL]**	5.3 (4.4–6.6)	5.6 (4.6–6.8)	5.5 (4.4–6.6)	0.345
**Platelet count [/nL]**	214 (177–258)	217 (175–263)	212.0 (166–266)	0.465

*Note:* Longitudinal course of copper metabolism parameters is depicted according to time on treatment: ‘therapy‐naïve’ (≤ 1 year of treatment), ‘short‐term’ (> 1–5 years of treatment), ‘median‐term’ (> 5–10 years) and ‘long‐term’ (> 10 years) of WD treatment. Data are shown as Median (IQR) if not stated otherwise.

Additionally, we analysed whether CuEXC values at T0 showed differences depending on the respective therapy regimen (median doses) and different subcohorts (stable vs. unstable; short−/median‐ and long‐term). No clinically meaningful differences were detected as IQRs across treatment groups were highly overlapping (Table S3). Finally, we assessed whether CuEXC differed by clinical phenotype (hepatic vs. neurological/mixed; Figure S1). No significant differences were observed in any subgroup (short‐term: *p* = 0.790; median‐term: *p* = 0.330; long‐term: *p* = 0.155). Although CuEXC tended to be higher in neurological phenotypes early after diagnosis, this pattern did not persist during longer follow‐up. In two therapy‐naïve patients with neurological symptoms, baseline CuEXC levels were markedly elevated > 2.08 μmol/L (i.e., 2.88 and 3.11 μmol/L, respectively).

## 4 Discussion

The two main challenges in treating patients with WD are timely and accurate initial diagnosis and ensuring a well‐balanced copper homeostasis for patients under chelation or zinc therapy. The newly emerged copper parameters REC and CuEXC have been proposed to fulfil these tasks, however, real‐world data in large cohorts with WD are scarce and those available are conflicting. In this large mixed paediatric and adult cohort, we reaffirm that REC is an excellent diagnostic biomarker for WD, outperforming both ceruloplasmin and total serum copper. Our data support its incorporation into routine clinical work‐up of suspected WD, in line with the recommendations of the 2025 EASL‐ERN Clinical Practice Guidelines [10]. Recently, Djebrani‐Oussedik et al. and Guillaud et al. reported REC to be an accurate and robust biomarker for initial diagnosis of WD in a French cohort at a cut‐off level of > 14% with very high sensitivity and specificity, especially for treatment naïve patients [19, 21].

Lorenzen et al. found similar results in a slightly smaller, combined Spanish‐Danish cohort and proposed to include the REC into an updated version of the Leipzig score in the future [20]. With an AUC of 0.955, our findings align with and strengthen evidence from these cohorts, while substantially expanding the available data for adult patients. Of note, in our cohort, the calculated optimal threshold for distinguishing WD from non‐Wilsonian liver disease was even lower at 10.6%. This might at least in part be explained by the large proportion of pre‐treated patients in our cohort, with > 97% of included patients being on chelation treatment at the time of the first available REC value. Therefore, higher cut‐offs (> 14%) may be more appropriate in newly diagnosed, untreated populations. All genetically confirmed WD patients with REC below 10.6% had been treated for over a decade and were already diagnosed during childhood. Contrastingly, in our study, all six therapy‐naiive WD patients showed a REC > 15%. Several studies have shown that REC is not a sufficient parameter for disease monitoring as it usually declines during long‐term chelation therapy and fails to assess subtle changes in copper metabolism [24, 25, 28]. Importantly, REC retained its diagnostic accuracy across diverse clinical settings. It has potential for broader application in screening efforts, both in paediatric and adult populations and across various medical specialties—such as neurology and psychiatry—with the goal of reducing diagnostic delays in WD. However, it remains unclear whether REC elevation alone is sufficient for initiation of chelating treatment, although it reduces potential delay of WD diagnosis through genetic testing. Elevated REC values should still be confirmed within the clinical context due to potential errors in pre‐analytics [17].

Due to limited availability of REC and broad usage of Cp and total serum copper, we also assessed their potential as initial screening parameters for WD using ROC analysis. Previously, the most accurate threshold for diagnosis of WD for serum ceruloplasmin was reported to be below 14 mg/dL (sensitivity 93% and specificity 100%) in a series of 57 adults and children with WD with liver dysfunction and/or neurological deficits [29], and below 20 mg/dL (sensitivity 95% and specificity 84.5%) in a series of 40 clinically asymptomatic children with elevated serum transaminases [30]. The ceruloplasmin threshold identified in our cohort (< 16.5 mg/dL) aligns closely with the ranges described in these earlier studies. Overall, ceruloplasmin shows a good diagnostic performance with an AUC comparable to REC and total copper, although with somewhat lower specificity. Of note, a Leipzig score ≥ 4 incorporates ceruloplasmin as diagnostic component. Consequently, its association with final WD diagnosis is not independent and must therefore be interpreted cautiously. Total serum copper is not commonly used in the diagnostic work‐up of WD, although levels are often reduced due to the typically decreased ceruloplasmin concentrations [10]. Interestingly, ROC analysis provided a high AUC of 0.842 for total Cu at a ≤ 9.55 μmol/L cut‐off, very similar to the findings of Lorenzen et al. [20] with a cut‐off ≤ 9.3 μmol/L. Because total copper measurement is more widely available than REC, it may be considered for diagnostic purposes; however, it should be interpreted with caution in ALF and conditions of malassimilation [2], where total copper levels are lower than in other diseases. It should also be noted that other diseases apart from WD can cause ceruloplasmin (and, subsequently, copper) depletion [26, 31].

A key contribution of this study is the evaluation of REC in adult ALF, a scenario in which the routine diagnostic components of the Leipzig score are often unavailable or unreliable [22]. In this critically ill subgroup, REC clearly distinguished WD‐associated ALF from other etiologies. These results extend the observations from the paediatric cohort by Spirea et al. [22] and indicate that REC is a highly valuable rapid biomarker for fulminant copper toxicity. Timely discrimination between WD‐ALF and non‐WD causes is crucial, as it directly affects decisions regarding plasma exchange, medical stabilization or urgent liver transplantation. Our findings therefore suggest that REC should be incorporated into early diagnostic algorithms for adults presenting with ALF of unclear origin, although the ALF subgroup includes only two WD cases. Larger multicenter studies are therefore needed to confirm the diagnostic performance of REC in adult ALF.

Measuring CuEXC requires only a simple ultrafiltration step followed by direct copper quantification, allowing results to be obtained as quickly as other copper parameters. Notably, CuEXC offers a reliable and immediately interpretable estimate of NCC, eliminating the problem of misleading or negative calculated NCC values [16, 18, 19].

Our longitudinal analysis demonstrates that CuEXC and UCE provide several important insights that refine the interpretation of these biomarkers. First, treatment‐naïve patients show the expected robust biochemical response, with a marked decline in CuEXC and UCE over the first year of chelation, supporting CuEXC as a sensitive marker of initial copper mobilization and treatment onset. Second, in very stable, long‐term treated patients, CuEXC and UCE remain consistently low with narrow interquartile ranges and minimal intraindividual variability, indicating excellent and durable metabolic control. Third, however, despite these clear within‐patient dynamics, the ability of CuEXC to discriminate between clinically stable and unstable patients is limited, as group‐level differences remain small and statistically non‐significant over time. Taken together, CuEXC demonstrates particularly robust discrimination between spectrum endpoints (therapy‐naïve and very stable patients), but only subtle separation at best in intermediate stable/unstable groups. UCE revealed more pronounced results in these subgroups and hence remains the established clinical reference standard, while CuEXC emerges as complementary biomarker. UCE off‐treatment (48 h pause) served as a key component of our stability classification, with approximate target ranges of < 1.5 μmol/d (optimal), 1.5–2.5 μmol/d (acceptable), > 2.5 μmol/d (poor control) based on European guidelines [10] and recent comparative data [13, 14]. This conservative classification may contribute to overlap between stable/unstable groups; however, it reflects a common challenge in heterogenous real‐world cohorts. Our findings suggest that both CuEXC and UCE are particularly useful for monitoring early treatment response and long‐term biochemical stability at the individual level, while their role as cross‐sectional classifiers of clinical stability appears restricted, underscoring the need to interpret these biomarkers in conjunction with clinical context, additional measures of copper status, and other laboratory results.

The refined subgroup analyses further validate the biphasic CuEXC trajectory observed by Ngwanou et al. [24], with dramatic initial declines in therapy‐naïve patients, contrasting with stable maintenance levels in very stable long‐term patients and subtle uptrends in broader long‐term cohorts. Our data also show an uptrend in CuEXC after initial treatment decline, particularly in our long‐term cohort, alongside consistently higher UCE values in long‐term versus short‐term patients. This paradoxical pattern during maintenance therapy likely reflects a combination of factors, including gradual adherence relaxation, cumulative chelator exposure with dose adjustments and possible remodelling of hepatic/extrahepatic copper pools toward a higher steady state. Clinically, these trends signal potential undertreatment in a subset of patients despite apparent clinical stability, emphasizing the need to avoid static biomarker targets and instead adopt time‐adapted CuEXC and UCE ranges to guide individualized management. Additionally, the longitudinal data demonstrate that early and consistent therapy in WD rapidly normalizes copper homeostasis, most strikingly in the therapy‐naïve subcohort with a pronounced reduction in CuEXC, UCE and NCC within the first year. In the short‐, medium‐ and long‐term treated subcohorts, CuEXC remains largely stable over time, suggesting sustained control of copper metabolism under long‐term therapy, whereas CuEXC cannot reliably discriminate stable from unstable patients across chelator therapies.

Moreover, we found no sustained differences in CuEXC values between hepatic and neurological/mixed phenotypes in contrast to earlier studies suggesting higher CuEXC in extrahepatic presentation. Poujois et al. previously reported elevated CuEXC levels in WD patients with neurological involvement and increased extrahepatic disease burden [32], suggesting that this biomarker may reflect not only hepatic copper overload but also more widespread systemic copper dysregulation. In our cohort, only treatment‐naïve patients with neurological involvement showed markedly elevated CuEXC levels > 2.08 μmol/L at diagnosis, which is associated with extrahepatic involvement and its severity as previously reported [17].

Taken together, our longitudinal analyses clarify complementary roles for copper biomarkers in WD management, with parallel trajectories of CuEXC and UCE across treatment phases. REC and CuEXC demonstrate strengths at spectrum endpoints: REC for rapid WD diagnosis, also in adult ALF and CuEXC for distinguishing therapy‐naïve copper overload (> 1.5 μmol/L) from optimal long‐term copper control, while UCE serves as the established clinical reference standard per guidelines for therapy monitoring under standardized conditions. These findings advocate harmonized, time‐adapted strategies that leverage each biomarker's unique contributions alongside clinical context and adherence assessment for comprehensive long‐term WD care.

The strengths of this study include the size and heterogeneity of the cohort, the systematic evaluation of REC and CuEXC across defined timepoints, and the focused assessment of adult ALF—a group notoriously difficult to diagnose. Our study employed a retrospective case–control approach (*n* = 299; 215 confirmed WD, Leipzig ≥ 4) comprising patients with clinically indicated REC/CuEXC measurements across outpatient, inpatient and ICU settings, representing a realistic diagnostic challenge scenario in a typical clinical hepatological setting. While enabling the largest reported REC evaluation, this design provides robust effect size but can either introduce spectrum effect and selection bias. Limitations include the retrospective design, small number of WD‐ALF patients (*n* = 2), variability in pretreatment duration, and potential inter‐laboratory differences in CuEXC methodology. UCE measurements in adults were performed after a 2‐day treatment cessation, which reflects local practice but may limit direct comparison to other cohorts. Moreover, clinical indication for testing likely enriched for higher disease probability, potentially overestimating performance relative to unselected populations. Case–control verification bias might complicate further interpretation, as WD diagnosis incorporates copper parameters (UCE, ceruloplasmin), creating incorporation circularity that inflates biomarker AUCs. Further prospective multicenter studies in consecutive undiagnosed liver disease cohorts are hence needed to validate REC performance under real‐world diagnostic conditions.

## 5 Conclusion

This study provides robust evidence that REC is a highly accurate, rapid and clinically valuable biomarker for diagnosing WD, including in adults with acute liver failure—an area in which diagnostic clarity is urgently needed. Furthermore, given its unexpectedly strong diagnostic value, ceruloplasmin in combination with total copper and UCE may serve as a useful adjunct in the initial diagnostic assessment—particularly in settings where REC analysis is currently unavailable. As with all individual markers, however, ceruloplasmin total copper should not be used in isolation and must be interpreted still within a comprehensive diagnostic framework. Overall, our findings support the consideration of REC in a potential future revision of the Leipzig score. During therapy monitoring, CuEXC demonstrates excellent discrimination between therapy‐naïve patients with copper overload and very stable long‐term patients, confirming its value for both initial response assessment and verification of optimal maintenance therapy.

CuEXC thus emerges as complementary to UCE for identifying patients needing adherence counselling or therapy optimization during maintenance therapy, underscoring the need for multicenter prospective studies to standardize CuEXC methodology and to refine monitoring strategies and thresholds for disease management.

## Author Contributions

Study concept and design: I.M. Acquisition, analysis and interpretation of the data: S.K., A.R., S.W., J.L., A.L. and I.M. Drafting the article: S.K. and I.M. Critical revision for important intellectual content: S.K., H.Z., M.N., T.L., A.F., P.M. and I.M. Statistical analysis: S.K., H.Z., M.N., A.F. and I.M. Study supervision: I.M. All authors approved the final version of the article and agreed to be accountable for all aspects of the work.

## Funding

For the publication fee, we acknowledge financial support by Heidelberg University.

## Ethics Statement

The retrospective data collection was approved by the Ethics Committee of the University of Heidelberg (protocol number: S‐214/2025), which complied with the provisions of the Good Clinical Practice guidelines and the Declaration of Helsinki and local laws and fulfilled Regulation (EU) 2016/679 of the European Parliament and the Council of 27 April 2016 on the protection of natural persons with regard to the processing of personal data (ID number DSAN854‐A‐OS/5). All patients provided informed consent for this study.

## Conflicts of Interest

S.K., A.R., H.Z., S.W., J.L., A.L., T.L., M.N. and A.F. declare no COI. PM received Honoria for lectures from AstraZeneca, Falk and Abbvie. I.M. advises for Univar, Orphalan and MetaLead, received travel grants from Univar, is Principal Investigator on sponsored studies with Univar and received speaker's fees from Univar and Orphalan.

## Supporting information


**Table S1:** Laboratory baseline characteristics only Wilson's disease patients among phenotype and paediatric sub‐cohort.
**Table S2:** Laboratory baseline characteristics of patients with acute liver failure (ALF).
**Table S3:** Median CuEXC values and corresponding daily doses according to treatment modality, clinical stability and treatment duration in adult Wilson's disease patients.
**Table S4:** Longitudinal course of exchangeable copper in Wilson's disease adult sub‐cohort for therapy‐naïve patients (≤ 1 year from initial diagnosis).
**Table S5:** Longitudinal course of exchangeable copper in Wilson's disease adult sub‐cohort during short‐term follow‐up (> 1 to 5 years from initial diagnosis).
**Table S6:** Longitudinal course of exchangeable copper in Wilson's disease adult sub‐cohort during median‐term follow‐up (> 5–10 years from initial diagnosis).
**Table S7:** Longitudinal course of exchangeable copper in Wilson's disease adult sub‐cohort during long‐term follow‐up (> 10 years from initial diagnosis).
**Figure S1:** Longitudinal course of exchangeable copper in Wilson's disease in neurological/mixed and hepatic patients.

## Data Availability

The data that support the findings of this study are available on request from the corresponding author. The data are not publicly available due to privacy or ethical restrictions.
