# Supplementary material for: Relative Exchangeable and Exchangeable Copper: Emerging New Biomarkers for Diagnosis and Therapy Monitoring in Wilson's Disease
Source: Liver Int. 2026 May 4;46:e70666. doi: 10.1111/liv.70666 (PMC13139700; doi:10.1111/liv.70666)
Supplement: Supplementary file 1 — Table S1: Laboratory baseline characteristics only Wilson's disease patients among phenotype and paediatric sub‐cohort. Table S2: Laboratory baseline characteristics of patients with acute liver failure (ALF). Table S3: Median CuEXC values and corresponding daily doses according to treatment modality, clinical stability and treatment duration in adult Wilson's disease patients. Table S4: Longitudinal course of exchangeable copper in Wilson's disease adult sub‐cohort for therapy‐naïve patients (≤ 1 year from initial diagnosis). Table S5: Longitudinal course of exchangeable copper in Wilson's disease adult sub‐cohort during short‐term follow‐up (> 1 to 5 years from initial diagnosis). Table S6: Longitudinal course of exchangeable copper in Wilson's disease adult sub‐cohort during median‐term follow‐up (> 5–10 years from initial diagnosis). Table S7: Longitudinal course of exchangeable copper in Wilson's disease adult sub‐cohort during long‐term follow‐up (> 10 years from initial diagnosis). Figure S1: Longitudinal course of exchangeable copper in Wilson's disease in neurological/mixed and hepatic patients. [file LIV-46-0-s001.docx]

**Supplemental Material:**

**Relative Exchangeable and Exchangeable Copper: Emerging new biomarkers for diagnosis and therapy monitoring in Wilson’s disease**

Sebastian Köhrer^1*^, Antoan Rusev^1*^, Holger Zimmer^2^, Silke Wolf^2^, Jessica Langel^1^, Andrea Langel^1^, Thomas Longerich^3^, Patrick Michl^1^. Moritz Niesert^4^, Alexander Fichtner^4^, Isabelle Mohr^1^

^1^Internal Medicine IV. Department of Gastroenterology. University Hospital Heidelberg. Heidelberg. Germany

^2^Internal Medicine I. Department of Endocrinology. diabetology. metabolic diseases and clinical chemistry. University Hospital Heidelberg. Heidelberg. Germany

^3^Institute of Pathology, Heidelberg University Hospital, Heidelberg, Germany

^4^Department of Pediatrics I. Center for Pediatrics and Adolescent Medicine. Medical Faculty Heidelberg. University Hospital Heidelberg. Heidelberg University. 69120 Heidelberg. Germany

*contributed equally

**Corresponding author:** PD Dr. med. Isabelle Mohr. Department of Gastroenterology. University Hospital Heidelberg. INF 410. 69120 Heidelberg. Phone: +49 6221 56 32818. eMail: [isabelle.mohr@med.uni-heidelberg.de](mailto:isabelle.mohr@med.uni-heidelberg.de)

**Supplemental Table 1: Laboratory baseline characteristics only Wilson’s disease patients among phenotype and pediatric sub-cohort**

| **Parameter**  **n=215 *total WD cohort** | **Hepatic Phenotype***  **n=164** | **Neurologic/Mixed Phenotype***  **n=51** | **p-value*** | **Pediatric sub-cohort**  **n=13** |
| --- | --- | --- | --- | --- |
| **Age (years)** | 38.9 (27.3- 50.8) | 41.5 (32.9-59.0) | 0.033 | 13,0 (9.2-1 6.1) |
| **Sex (female in %)** | 84 (51.2) | 22 (43.1) | 0.279 | 6 (46.1) |
| **Relative Exchangeable Copper [%]** | 18.40 (14.20- 43.30) | 22.25 (14.5-38.08) | 0.607 | 20.90 (16.05 -40.65) |
| **Exchangeable Copper [µmol/L]** | 0.92 (0.68-1.29) | 0.97 (0.66-1.24) | 0.487 | 0.97 (0.77- 1.18) |
| **24h Urinary copper excretion [µmol/d]** | 1.61 (0.99 -2.23) ** | 1.78 (1.09-3.13) ** | **0.030** | 5.54 (4.47 -9.26)*** |
| **Non-ceruloplasmin bound copper (NCC) [g/L] (calculated)** | 0.51 (0.01 -1.86) | 0.69 (0.20-1.40) | 0.400 | 0.82 (0.09-4.32) |
| **Ceruloplasmin [g/L]** | 0.10 (0.04-0.14) | 0.10 (0.05- 0.14) | 0.833 | 0.13 (0.08-0.27) |
| **Total serum copper [µmol/L]** | 4.95 (2.12-8.25) | 4.8 (2.0-8.0) | 0.696 | 5.20 (2.45-14.85) |
| **Aspartate aminotransferase [U/L]** | 29.0 (20.0 -42.0) | 24.0 (19.0 – 36.0) | **0.050** | 31.0 (21.0-72.0) |
| **Alanine aminotransferase [U/L]** | 46.5 (30.0- 66.0) | 34.0 (26.0 – 51.0) | **0.007** | 43.0 (22.0 -150.0) |
| **Gamma-glutamyl transferase [U/L]** | 34.0 (23.0-55.7) | 30.0 (22.0-52.0) | 0.735 | 26.0 (14.0-46.5) |
| **Alkaline phosphatase [U/L]** | 84 (70.5-113.5) | 91.0 (64.0 -107.0) | 0.785 | 247.0 (225.0 -285.0) |
| **Serum bilirubin [mg/dl]** | 0.7 (0.5- 1.1) | 0.7 (0.5-1.1) | 0.507 | 0.4 (0.3-0.8) |
| **International Normalized Ratio** | 1.03 (0.98-1.07) | 1.06 (1.01- 1.12) | **0.008** | 1.05 (0.99-1.08) |
| **White blood count [/nl]** | 5.62 (4.72 -6.89) | 5.6 (4.05- 6.62) | 0.285 | 5.79 (5.02 – 6.98) |
| **Platelet count [/nl]** | 175 (126- 234) | 234 (196 -270) | **0.001** | 315 (262- 363) |

*****p-value for hepatic vs. neurologic/mixed phenotype; **48h therapy cessation; ***on therapy

**Supplemental Table 2: Laboratory baseline characteristics of patients with acute liver failure (ALF)**

| **Parameter** | **All patients with ALF**  **N=29** | **ALF due to Wilson’s disease**  **N=2** | **ALF of non-Wilsonian etiology**  **N=27** | **p-value** |
| --- | --- | --- | --- | --- |
| **Age (years)** | 41.50 (35.62 – 53.58) | 36.87 (IQR 26.91) | 41.5 (36.0-53.67) | 0.399 |
| **Sex (female in %)** | 13 (44.82) | 2 (100%) | 11 (40.74) | 0.207 |
| **Relative Exchangeable Copper [%]** | 6.4 (4.5-8.2) | 42.15 (35.7) | 6.1 (4.4-7.6) | **0.005** |
| **Exchangeable Copper [µmol/L]** | 0.78 (0.51-1.06) | 5.5 (5.3) | 0.76 (0.51-1.0) | **0.020** |
| **24h Urinary copper excretion [µmol/d]** | 18.68 (1.28-63.92) | 63.92 (40.98) | 1.6 (0.96 – 1.6) *n=15 | 0.333 |
| **Non-ceruloplasmin bound copper (NCC) [g/L] (calculated)** | 0.68 (0.01-3.24) | 11.28 ( 9.98) | 0.58 (0.01-2.57) | 0.143 |
| **Ceruloplasmin [g/L]** | 0.23 (0.17- 0.23) | 0.08 (0.07) | 0.25 (0.20-0.34) | **0.010** |
| **Total serum copper [µmol/L]** | 11.8 (9.15-16.35) | 13.45 (10.9) | 11.8 (8.7- 16.5) | 0.768 |
| **Aspartate aminotransferase [U/L]** | 236 (72.5-811) | 264.0 (169.0) | 236.0 (68.0-871.0) | 0.966 |
| **Alanine aminotransferase [U/L]** | 144 (37.0-565.0) | 143.0 (136.0) | 144.0 (35.0-649.0) | 0.889 |
| **Gamma-glutamyl transferase [U/L]** | 102 (48.5-186.0) | 333.5 (143) | 144 (35.0-185.0) | 0.276 |
| **Alkaline phosphatase [U/L]** | 140 (106.25-203.54) | 117.5 (74 .0) | 140 (106.75-208.0) | 0.529 |
| **Serum bilirubin [mg/dl]** | 17.4 (9.1 -23.54) | 6.84 (5.34) | 18.35 (9.37-23.55) | 0.059 |
| **International Normalized Ratio** | 2.9 (1.9- 3.34) | 2.81 (2.75) | 3.0 (1.8 - 3.53) | 0.123 |
| **White blood count [/nl]** | 9.35 (6.47-12.43) | 12.39 (11.1) | 9.35 (6.2-11.17) | 0.276 |
| **Platelet count [/nl]** | 118 (49.0-245.0) | 185 /117) | 118 (46.0-238.0) | 0.542 |

*****p-value for ALF due to Wilson disease vs. ALF of Non-Wilsonian etiology

**Supplemental Table 3: Median CuEXC values and corresponding daily doses according to treatment modality, clinical stability, and treatment duration in adult Wilson’s disease patients**

| **Treatment** | **Median Daily Dose (IQR)** | **Median CuEXC µmol/L (IQR)** | **Median Daily Dose (IQR)** |
| --- | --- | --- | --- |
|  | Stable (n=26) | 1.2 (0.9–1.7) | 1200 mg (900–1200) |
|  | Unstable (n=22) | 1.3 (1.1–2.3) | 1500 mg (900–1500) |
|  | Short-term (n=14)* | 1.9 (1.4– 2.6) | 1200 mg (900–1200) |
|  | Median-term (n=12) | 1.4 (1.0–2.0) | 1300 mg (900–1400) |
|  | Long-term (25) | 1.1 (0.8–1.5) | 1100 mg (900–1200) |
| **Trientine (overall)** | Overall (n=103) | 1.3 (0.9–1.9) | 1200 (800–1400) for trientine dihydrochloride  750 (450-900) for trientine tetrahydrochloride |
|  | Stable (n=23) | 1.1 (0.8–1.6) | 1000 mg (800–1000) |
|  | Unstable (n=30) | 1.2 (0.9–2.2) | 1400 mg (1000–1600) |
|  | Short-term (n=2) | 1.8 (1.3-2.4) | 1200 mg (1000–1400) |
|  | Median-term (n=20) | 1.4 (1.0 -2.0) | 1300 mg (1000–1400) |
|  | Long term (n=81) | 1.1 (0.8-1.6) | 1100 mg (800–1200) |
| **Zinc acetate** | Overall (n=26) | 0.9 (0.6–1.2) | 150 mg (135–150) |
|  | Stable | NA** | NA** |
|  | Unstable | NA** | NA** |
|  | Short-term | NA** | NA** |
|  | Median-term | NA** | NA** |
|  | Long term (n=26)** | 0.9 (0.6–1.2) | (135–150) |
|  | Stable (n=26) | 1.2 (0.9–1.7) | 1200 mg (900–1200) |

*including also n=6 therapy-naïve patients; **all classified as very-stable. NA= not available.

**Supplemental Table 4: Longitudinal course of exchangeable copper in Wilson’s disease adult sub-cohort for therapy-naïve patients (≤1 year from initial diagnosis)**

| **Laboratory Parameter** | **WD sub-cohort at T0**  **n=6** | **WD sub-cohort at T1**  **n= 6** | **WD sub-cohort at T2**  **n=5** | **p-value** |
| --- | --- | --- | --- | --- |
| **Relative Exchangeable Copper [%]** | 45.0 (35.0–55.0) | 28.0 (20.0–35.0) | 22.0 (16.0–30.0) | **0.010** |
| **Exchangeable Copper [µmol/L]** | 1.80 (1.40–2.40) | 1.10 (0.80–1.40) | 0.90 (0.65–1.20) | **0.005** |
| **24h Urinary copper excretion [µmol/d]** | 3.20 (2.50–4.20) | 1.80 (1.20–2.60) | 1.50 (1.00–2.30) | **0.003** |
| **Non-ceruloplasmin bound copper (NCC) [g/L] (calculated)** | 1.20 (0.80–1.80) | 0.80 (0.40–1.40) | 0.60 (0.20–1.10) | **0.020** |
| **Ceruloplasmin [g/L]** | 0.06 (0.04–0.09) | 0.09 (0.07–0.12) | 0.10 (0.08–0.14) | **0.040** |
| **Total serum copper [µmol/L]** | 6.0 (3.5–8.5) | 5.0 (3.0–7.5) | 4.5 (3.0–6.5) | 0.080 |
| **Aspartate aminotransferase [U/L]** | 52.0 (38.0–78.0) | 32.0 (24.0–48.0) | 28.0 (22.0–40.0) | **0.020** |
| **Alanine aminotransferase [U/L]** | 68.0 (45.0–98.0) | 40.0 (28.0–72.0) | 35.0 (24.0–60.0) | **0.030** |
| **Gamma-glutamyl transferase [U/L]** | 55.0 (32.0–120.0) | 35.0 (22.0–80.0) | 28.0 (18.0–60.0) | 0.050 |
| **Alkaline phosphatase [U/L]** | 95.0 (70.0–135.0) | 90.0 (75.0–125.0) | 88.0 (70.0–120.0) | 0.400 |
| **Serum bilirubin [mg/dl]** | 1.6 (1.0–2.4) | 1.2 (0.9–1.8) | 1.0 (0.8–1.4) | 0.060 |
| **International Normalized Ratio** | 1.1 (1.0–1.3) | 1.0 (0.9–1.2) | 1.0 (0.9–1.1) | 0.150 |
| **WBC [/nl]** | 5.0 (3.8–6.8) | 5.2 (4.0–7.0) | 5.4 (4.2–7.5) | 0.700 |
| **Thrombocytes [/nl]** | 195 (120–260) | 215 (130–280) | 240 (150–300) | 0.200 |

**Supplemental Table 5: Longitudinal course of exchangeable copper in Wilson’s disease adult sub-cohort during short-term follow-up (>1 to 5 years from initial diagnosis)**

| **Laboratory Parameter** | **WD sub-cohort at T0**  **n=10** | **WD sub-cohort at T1**  **n= 10** | **WD sub-cohort at T2**  **n=9** | **p-value** |
| --- | --- | --- | --- | --- |
| **Relative Exchangeable Copper [%]** | 22.0 (16.5-32.0) | 19.5 (17.0-27.0) | 21.0 (16.0-30.0) | 0.520 |
| **Exchangeable Copper [µmol/L]** | 0.80 (0.55-1.10) | 0.60 (0.45-1.05) | 0.65 (0.50-1.15) | 0.680 |
| **24h Urinary copper excretion [µmol/d]** | 2.20 (1.70-4.20) | 1.10 (0.90-1.60) | 1.20 (0.60-2.40) | **0.045** |
| **Non-ceruloplasmin bound copper (NCC) [g/L] (calculated)** | 0.25 (0.05-0.80) | 0.22 (0.05-1.40) | 0.10 (0.02-0.45) | 0.280 |
| **Ceruloplasmin [g/L]** | 0.08 (0.05-0.14) | 0.08 (0.05-0.14) | 0.08 (0.04-0.15) | 0.650 |
| **Total serum copper [µmol/L]** | 3.8 (2.5-8.0) | 3.6 (2.4-8.2) | 4.0 (2.0-7.2) | 0.580 |
| **Aspartate aminotransferase [U/L]** | 28.0 (18.0-38.0) | 26.0 (16.0-45.0) | 27.0 (21.0-37.0) | 0.180 |
| **Alanine aminotransferase [U/L]** | 42.0 (33.0-65.0) | 35.0 (22.0-85.0) | 39.0 (27.0-76.0) | 0.420 |
| **Gamma-glutamyl transferase [U/L]** | 30.0 (19.0-65.0) | 27.0 (20.0-77.0) | 24.0 (19.0-48.0) | 0.380 |
| **Alkaline phosphatase [U/L]** | 80.0 (56.0-105.0) | 81.0 (68.0-106.0) | 80.5 (66.0-103.0) | 0.720 |
| **Serum bilirubin [mg/dl]** | 1.1 (0.8-1.7) | 1.1 (0.8-1.4) | 1.0 (0.8-1.2) | 0.950 |
| **International Normalized Ratio** | 1.0 (0.9-1.1) | 1.0 (0.9-1.1) | 1.0 (0.9-1.1) | 0.980 |
| **WBC [/nl]** | 5.3 (4.0-6.8) | 5.4 (4.2-6.2)\| | 5.7 (4.2-7.4) | 0.650 |
| **Thrombocytes [/nl]** | 225 (135-265) | 225 (115-280) | 268 (100-292) | 0.260 |

*p-value for T0 vs. T2

**Supplemental Table 6: Longitudinal course of exchangeable copper in Wilson’s disease adult sub-cohort during median-term follow-up (>5-10 years from initial diagnosis)**

| **Laboratory Parameter** | **WD sub-cohort at T0**  **n=32** | **WD sub-cohort at T1**  **n= 32** | **WD sub-cohort at T2**  **n=23** | **p-value** |
| --- | --- | --- | --- | --- |
| **Relative Exchangeable Copper [%]** | 18.6 (10.3-27.4) | 16.1 (16.1- 34.9) | 20.4(11.6- 45.6) | 0.927 |
| **Exchangeable Copper [µmol/L]** | 0.84 (0.55-1.21) | 0.94 (0.64- 1.14) | 0.73 (0.60- 1.05) | 0.330 |
| **24h Urinary copper excretion [µmol/d]** | 1.06 (0.51-2.10) | 1.14 (0.75 -1.89) | 1.54 (0.65-2.32) | **0.002** |
| **Non-ceruloplasmin bound copper (NCC) [g/L] (calculated)** | 0.71 (0.01- 1.63) | 0.66 (0.14-1.65) | 0.67 (0.06-2.09) | 0.609 |
| **Ceruloplasmin [g/L]** | 0.1 (0.06 -0.16) | 0.1 (0.05-0.16) | 0.08 (0.02-0.16) | 1.0 |
| **Total serum copper [µmol/L]** | 4.5 (2.1 -8.5) | 4.90 (2.15-8.65) | 3.5 (1.65- 8.50) | 0.363 |
| **Aspartate aminotransferase [U/L]** | 23.0 (14.5-36.0) | 29.0 (18.2- 39.5) | 35.0 (23.0- 51.0) | 0.145 |
| **Alanine aminotransferase [U/L]** | 34.0 (25.0-56.5) | 44.5 (26.5 -69.0)) | 43.0 (31.0 – 71.0) | 0.133 |
| **Gamma-glutamyl transferase [U/L]** | 27.0 (17.0-64.0) | 28.0 (20.0 -51.0) | 33.0 (19.0- 61.0) | 0.233 |
| **Alkaline phosphatase [U/L]** | 79.0 (64.0-109.0) | 75.0 (63.0- 95.5) | 82.0 (74.0-94.0) | 0.789 |
| **Serum bilirubin [mg/dl]** | 0.7 (0.5- 1.0) | 0.7 ((0.5- 1.1) | 0.8 (0.6-1.2) | 0.899 |
| **International Normalized Ratio** | 1.0 (0.9-1.1) | 1.0 (1.0- 1.1) | 1.1 (1.0- 1.1) | 0.987 |
| **WBC [/nl]** | 5.8 (4.4- 7.1) | 5.4 (4.6- 6.5) | 5.5 (4.3- 7.0) | 0.679 |
| **Thrombocytes [/nl]** | 196 (148- 240) | 190 (158- 256) | 181 (111- 248) | 0.267 |

*p-value for T0 vs. T2

**Supplemental Table 7: Longitudinal course of exchangeable copper in Wilson’s disease adult sub-cohort during long-term follow-up (>10 years from initial diagnosis)**

| **Laboratory Parameter** | **WD sub-cohort at T0**  **n=132** | **WD sub-cohort at T1**  **n= 132** | **WD sub-cohort at T2**  **n=83** | **p-value** |
| --- | --- | --- | --- | --- |
| **Relative Exchangeable Copper [%]** | 20.75 (15.23-42.78) | 24.70 (15.47- 41.27) | 22.3 (14.2- 39.0) | 0.565 |
| **Exchangeable Copper [µmol/L]** | 0.98 (0.73-1.32) | 1.06 (0.77-1.31) | 1.08 (0.80 -1.37) | 0.155 |
| **24h Urinary copper excretion [µmol/d]** | 1.76 (1.16 -2.50) | 1.69 (1.04- 2.75) | 1.72 (1.17 - 2.52) | **0.002** |
| **Non-ceruloplasmin bound copper (NCC) [g/L] (calculated)** | 0.66 (0.16-1.14) | 0.74 (0.14-1.48) | 0.42 (0.01-1.11) | **0.016** |
| **Ceruloplasmin [g/L]** | 0.08 (0.03-0.14) | 0.09 (0.03-0.14) | 0.11 (0.04-0.15) | **0.002** |
| **Total serum copper [µmol/L]** | 4.9 (2.0-7.1) | 4.8 (1.93-7.60) | 5.5 (2.05 -7.83) | 0.690 |
| **Aspartate aminotransferase [U/L]** | 29.0 (22.0-37.0) | 31.5 (25.0-40.0) | 34.0 (25.3- 46.75) | 0.354 |
| **Alanine aminotransferase [U/L]** | 45.0 (30.0-64.0) | 47.5 (34.3-80.8) | 45.0 (33.5-76.5) | 0.876 |
| **Gamma-glutamyl transferase [U/L]** | 37.0 (26.0 -56.75) | 39.0 (28.0-58.0) | 28.0 (24.0-57.0) | 0.187 |
| **Alkaline phosphatase [U/L]** | 85.0 (69.0-106.0) | 95.0 (90.0-114.8) | 91.0 (75.0-109.5) | 0.456 |
| **Serum bilirubin [mg/dl]** | 0.74 (0.56-1.03) | 0.82 (0.55- 1.11) | 0.82 (0.55-1.14) | 0.110 |
| **International Normalized Ratio** | 1.03 (0.99-1.08) | 1.05 (1.0-1.10) | 1.05 (1.0-1.1) | 0.104 |
| **WBC [/nl]** | 5.39 (4.49-6.68) | 5.46 (4.51- 6.47) | 5.53 (4.48-6.72) | 0.657 |
| **Thrombocytes [/nl]** | 217.0 (182.0-263.0) | 213.0 (173.50 – 263.75) | 212 (171.0 – 269.5) | 0.345 |

*p-value for T0 vs. T2

**Supplemental Figure 1:** **Longitudinal course of exchangeable copper in Wilson’s disease in neurological/mixed and hepatic patients**


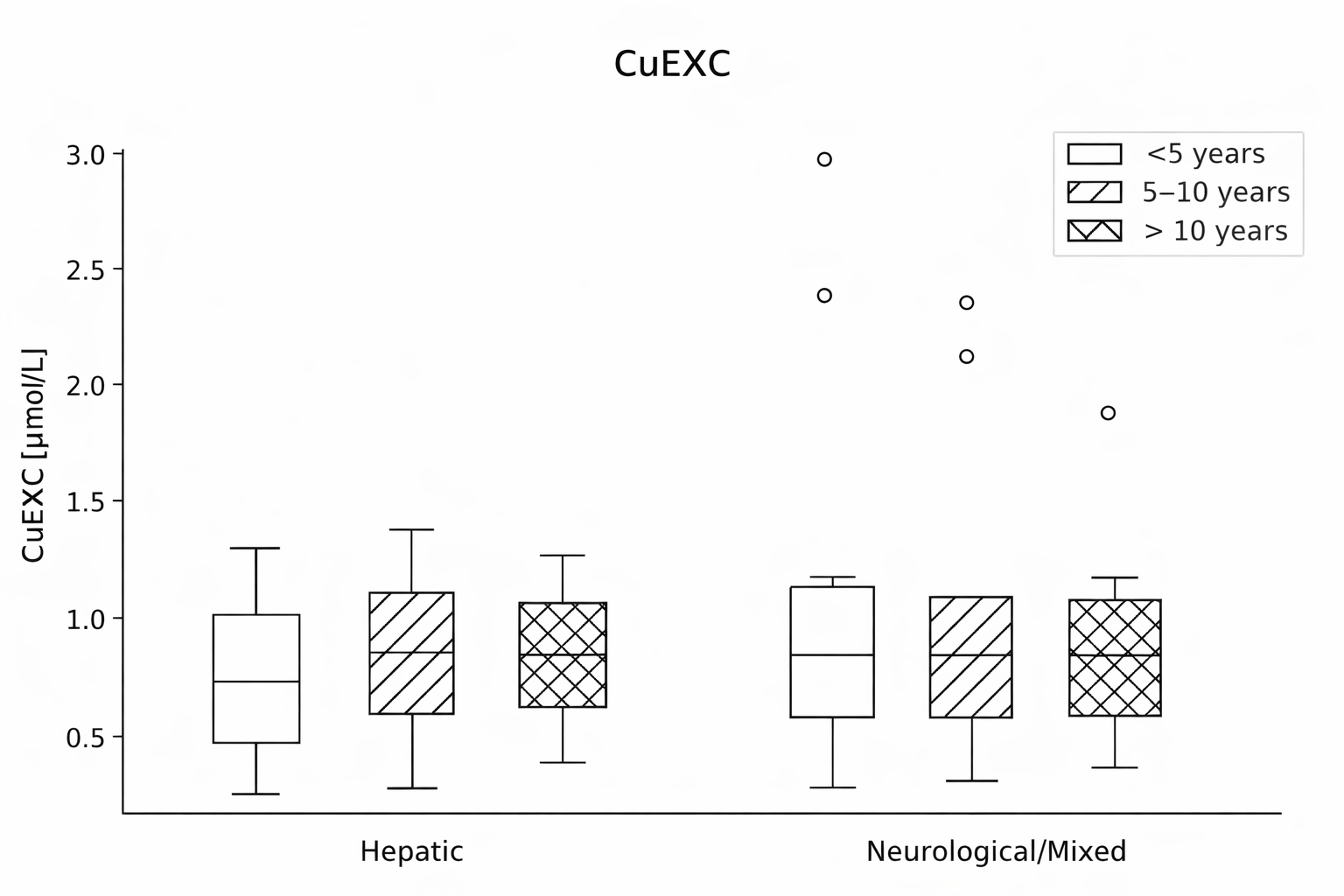


**Figure Legend:** Longitudinal course of exchangeable copper in Wilson’s disease in neurological and non-neurological patients. Median exchangeable copper values of WD patients with hepatic (left) vs. neurological/mixed disease phenotype (right). Data presented as box plots for short- (white fill, no hatching), median- (white fill, diagonal hatching), and long-term (white fill, cross-hatching) observational period of 12 months. Levels of significance: *p < 0.05; ns, not significant (Kruskal Wallis test).

**Supplemental information on Methods**

***Grouping definitions for WD subcohorts in monitoring CuEXC***

Additional conditions for grouping within the WD adult cohort were the following:

1. “Stable patients” were under unchanged therapy for ≥12 months with normal/mildly abnormal LFTs (comorbidities permitted) <2x of the upper limit of normal (ULN) and UCE ≤2.5 µmol/d after 48h treatment cessation with no clinical evidence of progressive hepatic, neurological, or psychiatric involvement attributable to WD during the last 5 years.
2. “Unstable patients” were requiring dose modification due to insufficient copper control (UCE >2.5 µmol/d, rising transaminases >2x ULN, clinical evidence of disease worsening) during the last 12 months after at least one year of previous treatment.
3. "Very stable" patients were defined as clinically stable for >5 years, had to have normal LFTs and UCE <1.6 µmol/d and needed no dose adjustments ≥24 months.
4. “Therapy-naïve patients” had not received any specific anti-copper therapy prior to the first available measurement (T0).

Overtreatment was defined as the presence of clinical or laboratory signs (e.g. cytopenia) compatible with excessive negative copper balance. We defined insufficient control as UCE >2.5 µmol/d in treated WD and interpreted in conjunction with clinical findings (e.g. LFT > 2-3x ULN, clinical deterioration). Patients with abnormal LFTs (>2x ULN) but UCE within target were evaluated case by case. Where abnormalities could be confidently attributed to non‑WD etiologies (e.g. metabolic liver disease, alcohol, drugs), patients were classified as stable; otherwise, they were considered unstable. Patients with normal LFTs, adequate therapy adherence, but UCE above target were classified as unstable, reflecting possible biochemical undertreatment. Classification as stable vs. unstable was assessed at each time point (t0, t1, t2).

***Exchangeable copper (CuEXC) and REC Determination***

For CuEXC determination. blood samples (serum) were collected in S-Monovette® Serum CAT 7.5ml (Sarstedt) and instantaneously transferred to our laboratory to be treated within 30 min. Blood was centrifuged at 1200G for 10 min and serum was prepared immediately for ultrafiltration. A two-step method (ultrafiltration-determination) was carried out to determine CuEXC by using EDTA as a chelator of high-copper-affinity. Sample extraction: To 1 ml of serum. add 1 ml of extraction solution (3.015 g of Titriplex III dissolved in 1000 ml of 0.9% NaCl solution). Subsequently. vortex the sample for 20 seconds. After an incubation period of 1 hour. transfer the sample to an ultracentrifugal filter (Amicon-Ultra-4. 30 kDa). After centrifugation for 20 minutes at 1200 G (3000 RPM with a 12 cm radius centrifuge). remove the filter and determine the copper content using the formula REC (%) = CuEXC [µmol/l]/ total serum copper [µmol/l] x 100. The measurements of copper in ultrafiltrates were performed by Zeeman AA 240 Z graphite furnace atomic absorption spectrometry. 40 μL of sample was injected into the furnace. Calibration for copper is performed with a standard solution (0.47 µmol/l). from which the device automatically creates two further dilutions (1:1.43 (0.329 µmol/l) and 1:3.3 (0.141 µmol/l)). Calibration is done at the beginning of each measurement series. The zero point (CAL ZERO) is determined at the start of calibration using a blank solution (extinction ≤ 0.02). The blank solution and the copper standards 1 – 4 are measured in triplicate. and the results are averaged. Calibration is calculated from these averages. The deviations of the triplicate standards must not exceed 20%. External calibration was conducted for copper at 327.4 nm.
